# Functions, accumulation, and biosynthesis of important secondary metabolites in the fig tree (*Ficus carica*)

**DOI:** 10.3389/fpls.2024.1397874

**Published:** 2024-07-02

**Authors:** Yawen Wang, Ximeng Liu, Siyu Chen, Qingjie Wang, Biao Jin, Li Wang

**Affiliations:** College of Horticulture and Landscape, Yangzhou University, Yangzhou, China

**Keywords:** *Ficus carica*, secondary metabolite, anthocyanin, coumarin, biosynthesis

## Abstract

*Ficus carica* is an economically important horticultural plant. Due to its abundant secondary metabolites, *F. carica* has gained interest for its applications in medicine and as a nutritional supplement. Both external and internal factors affect the accumulation of secondary metabolites in *F. carica*. The assembly of the *F. carica* genome has facilitated functional analysis of key genes and transcription factors associated with the biosynthesis of secondary metabolites, particularly anthocyanin. In this review, we summarize the various types and functions of secondary metabolites, with a particular focus on flavonoids, coumarins, and terpenes. We also explore the factors influencing their biosynthesis and accumulation, including varieties, tissue, environmental factors (e.g., light), stresses (e.g., high temperature, low temperature, drought, nutrient deficiencies, salinity), hormonal treatments, and developmental factors. Furthermore, we discuss the involvement of structural genes and transcription factors in the biosynthesis of secondary metabolites, specifically anthocyanin and furanocoumarins, knowledge of which will promote the breeding and genetic engineering of novel *F. carica* varieties.

## 1 Introduction

*Ficus carica*, commonly known as the fig tree, is one of the oldest cultivated plants and is among the most important commercial fruit trees in the genus *Ficus*. *F. carica* is extensively cultivated in numerous regions worldwide, particularly in nations of the Mediterranean Basin such as Portugal and Turkey (Dueñas et al., 2008). Fresh and dried *F. carica* fruits are nutritious sources of secondary metabolites, making them popular ingredients in culinary preparations such as sauces, fruit wines, and dried fruit assortments. Extracts of *F. carica* leaves and fruits, as well as latex, have been used to treat gastrointestinal, respiratory, inflammatory, metabolic, and cardiovascular conditions (Mawa et al., 2013). The accumulation of secondary metabolites in *F. carica* is influenced by internal and external factors, such as light quality, high temperature, drought, salt stress, and stress-associated hormones. Development, storage, and processing techniques also affect the accumulation of secondary metabolites in *F. carica* fruit (Sandhu et al., 2023). The availability of the genome of *F. carica* and advancements in molecular biology and sequencing technologies have yielded insights into the biosynthetic mechanisms of important secondary metabolites in *F. carica* (Usai et al., 2020). A comprehensive understanding of secondary metabolites in *F. carica* is essential for its horticultural cultivation, commercial significance, and potential health benefits. Here, we review the types and functions of secondary metabolites in *F. carica*, as well as the factors influencing their biosynthesis and accumulation. We also summarize the regulatory mechanisms of important secondary metabolites, including anthocyanins and furanocoumarins, in *F. carica*, focusing on the related structural genes and transcription factors.

## 2 Types and functions of secondary metabolites in *F. carica*

**Figure 1:**
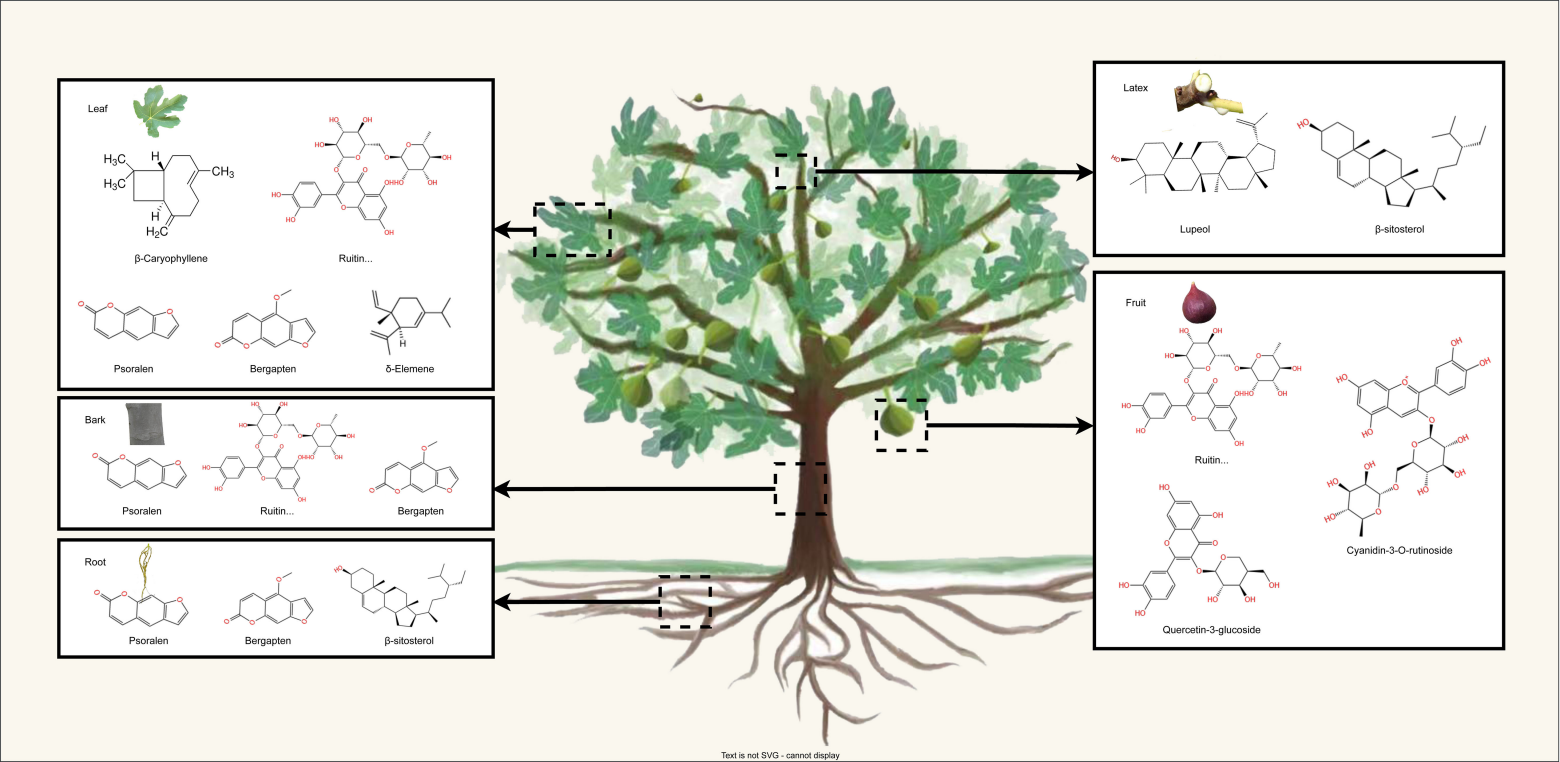
The schematic representation illustrates the distribution of main secondary metabolite in the leaves, fruits barks, roots, and latex of *F. carica*.

More than 100 bioactive compounds have been identified in *F. carica*. Almost all parts of the *F. carica* tree are abundant in secondary metabolites, particularly flavonoids, terpenes, and coumarins (Hajam and Saleem, 2022) (**Figure 1**). In this section, we focus on these secondary metabolites and their effects on health.

### 2.1 Flavonoids

Flavonoids, a class of polyphenolic secondary metabolites, are formed through the combination of two six-carbon phenyl rings connected by an oxygen heterocycle-containing ring. *F. carica* produces flavonoids in various organs, and the latex secreted from the vascular bundles of the petioles and peduncles also contains flavonoids and phenolic acids (Abdel-Aty et al., 2019). Flavonoids encompass several types, including anthocyanins, flavonols, and isoflavones, with significant research focusing on anthocyanins in *F. carica* fruit (Mao et al., 2023). The content and composition of these anthocyanins play a crucial role in determining the fruit’s color (Castañeda-Ovando et al., 2009). Anthocyanin pigments such as cyanidin, delphinidin, peonidin, petudinin, and malvidin are found in red, purple, and other dark-colored fruits.

Cyanidin is the predominant anthocyanin found in *F. carica*. During the ripening process of purple peel, cyanidin O-malonylhexoside levels increase dramatically. Other cyanidins, including cyanidin 3-O-glucoside and cyanidin-3, 5-O-diglucoside, also experience a significant increase (Wang et al., 2019). Among five different varieties of *F. carica* with distinct peel colors, cyanidin 3-rutinoside was the predominant anthocyanin, accounting for 48–81% of peel content and 68–79% of pulp content, while cyanidin 3-glucoside constituted 5–18% of peel content and 10–15% of pulp content (Dueñas et al., 2008). Notably, the dark-purple peel of the ‘Soltani’ variety contains nine identified anthocyanins, primarily cyanidin 3-rutinoside and cyanidin 3,5-diglucoside. The peel of ‘Soltani’ also revealed the presence of a novel rutinoside and petunidin dimer, yet its function remains unclear (Ammar et al., 2015).

Quercetin, an abundant flavonol in nature, has been extensively studied for its antioxidative and anti-inflammatory properties. *In vitro*, animal, and clinical studies have shown that quercetin can effectively reduce oxidative stress and exhibit anti-inflammatory activity (Ginwala et al., 2019; Jucá et al., 2020). Notably, quercetin has been found to reduce blood glucose levels in diabetic mice (Deepa et al., 2018; Dureshahwar et al., 2019). It is hypothesized that quercetin, along with other compounds, contributes to the observed hypoglycemic effect of the extract. Rutin, a glycoside form of quercetin, is predominantly present in *F. carica* fruit and leaves. Rutin has significant antioxidant activity, anticancer activity, and anti-inflammatory and antibacterial effects (Yang et al., 2008; Hao et al., 2016). It is a popular dietary supplement without any significant side effects. According to the available reports, the highest extraction rate of rutin from *F. carica* pulp was 0.44% (Ammar et al., 2015). In addition to flavonols like quercetin and rutin, *F. carica* also contains isoflavones. Isoflavones, which are 3-phenyl derivatives of benzone rings, are synthesized during the phenylalanine metabolism in plants. *F. carica* fruit has been found to contain 16 types of isoflavones, while the leaf contains 2 types. Interestingly, the isoflavones present in *F. carica* fruit have been shown to possess anti-inflammatory effects by inhibiting the production of nitric oxide (NO) (Liu et al., 2019). Several specific isoflavones, including ficucaricone A, ficucaricone B, and indicanine A, have been reported to exhibit potent anti-inflammatory activities (Magalhães et al., 2006).

### 2.2 Coumarins

Coumarins account for 51.46% of the volatile oils in *F. carica* leaves (Shahrajabian et al., 2021). Psoralen, bergapten, angelicin, rutaretin, pimpinellin, and seselin are all members of the coumarin family, serving as important secondary metabolites in *F. carica* (Marrelli et al., 2012). Among them, psoralen and bergapten are two of the most prominent coumarins due to their medicinal importance. They have been identified as anticancer agents with the ability to suppress the survival and migration of triple-negative breast cancer MDA-MB-231 cells (Zhang et al., 2018). Psoralens have shown promise in clinical studies, upregulating the level of ERα protein in osteoclasts and inhibiting the growth of breast cancer cells (Zhang et al., 2017). Additionally, psoralens and bergaptens can inhibit acetylcholinesterase (AChE) activity and play a role in the treatment of Alzheimer’s disease (Parhiz et al., 2015). Due to their ability to interact with DNA when exposed to ultraviolet A radiation (UV-A), these compounds, especially psoralens, are often used in the treatment of skin diseases such as vitiligo, alopecia areata, psoriasis, and neodermal diseases (Son et al., 2017). Psoralens and bergaptens have also demonstrated significant insecticidal activity properties (Chung et al., 2011; Siyadatpanah et al., 2022).

### 2.3 Terpenes

*F. carica* is rich in terpenes. Carotenoids, which belong to the group of tetraterpenoids, are a large group of phytochemicals with powerful antioxidant activities (Kaulmann and Bohn, 2014). *F. carica* contains the carotenoids typically present in human plasma, including β-carotene, lutein, and lycopene (Su et al., 2002). Similar to anthocyanins, carotenoids contribute to the coloration of *F. carica* fruits, giving them an orange or yellow color. The carotenoid contents of fresh and dried fruits differ. B-carotene, zeaxanthin, and lutein are predominant in fresh *F. carica* and β-cryptoxanthin and lutein in dried *F. carica* (Arvaniti et al., 2019). These carotenoids can prevent cardiovascular disease by reducing blood pressure, preventing oxidative stress, and decreasing the secretion of pro-inflammatory factors (Mordente et al., 2011).

Triterpenoids and sesquiterpenes are abundant in the extracts of *F. carica* root, leaf, and latex. Dried and young *F. carica* fruits contain the triterpenoid 9,19-cyclopropyl-24,25-ethylene-5ene-3β-spirosterol, which has anticancer activity (Yin et al., 1997). The main sesquiterpenes in *F. carica* leaf are genanthene, β-caryophyllene, and τ-elemiene (Oliveira et al., 2010c). β-Caryophyllene is used in cosmetics for its spicy aroma (Reinsvold, 2010). Sesquiterpenes are also the most abundant terpenes in *F. carica* latex, accounting for 91% of all identified compounds.

Fresh latex from *F. carica* has been used to treat a variety of types of cancers (Oliveira et al., 2010b; Arvaniti et al., 2019).

## 3 Accumulation and distribution of secondary metabolites in *F. carica*

### 3.1 Varieties (cultivars)

The accumulation and distribution of secondary metabolites vary markedly among *F. carica* cultivars (**Table 1**). In general, dark *F. carica* varieties exhibit higher polyphenol content and total anthocyanin content. Compared with those with light-colored peels, such as green and yellowish-green peels, cultivars with black and purple peels have higher levels of phenolics, flavonoids, and anthocyanins (Çalişkan and Polat, 2011; Ercişli et al., 2012; Hssaini et al., 2020). ‘Bursa siyahi’ (purple) *F. carica* shows a much higher total proanthocyanidin content than ‘Sarilop’ (yellow) *F. carica* (yellow) (Kamiloglu and Çapanoğlu, 2015). However, some light-colored *F. carica* varieties exhibit slightly higher proanthocyanidin content (Hssaini et al., 2020). Additionally, varieties of different colors reportedly share some similar anthocyanin profiles, principally cyanidin-3-O-rutinoside (> 90% of total anthocyanins). Moreover, varieties with black peels have high levels of total phenolic compounds, and those with green, greenish-yellow, and brown fruit peels have high levels of chlorogenic acid, vitamin C, and total tannins (Solomon et al., 2006; Pereira et al., 2017).

**Table 1:** Advantageous secondary metabolites in the main cultivated *F. carica* varieties in fruit peel.

Country	Varieties	References	Advantageous Secondary Metabolites
**Israel**	Mission(Black)	(Solomon et al., 2006)	Total anthocyanin content
**Italy**	Mattalona (Black) Dottato	(Del Caro and Piga, 2008) (Russo et al., 2014)	Flavonols (rutin, anthocyanins, hydroxycinnamic acids) Total phenolic content Cyaniding-3-rutinoside
**Turkey**	Bursa siyahi(purple) Siyah 5	(Kamiloglu and Çapanoğlu, 2015) (Çalişkan and Polat, 2011)	Total flavonoid content Total proanthocyanidin content Total anthocyanin content Flavan-3-ols Total phenolic content Total anthocyanin content
**Albania**	Kraps Zi (dark)	(Hoxha and Kongoli, 2016)	Total phenolic content Total flavonoid content Total anthocyanin content
**Tunisia**	Soltani (purple) Kohli	(Ammar et al., 2015) (Harzallah et al., 2016)	Total phenolic content Total anthocynin content Prenylhydroxygenistein Total phenolic content Total tannis content
**Iran**	Sabz (green) Siyah (dark purple)	(Maghsoudlou et al., 2017)	Total phenolic content Total flavonoid content
**Spain**	Banane VB1 Cuello de Dama	(Pereira et al., 2017) (Vallejo et al., 2012) (Dueñas et al., 2008)	Total Vitamin C Total phenolic content Total anthocynins Luteolin 6C-hexose-8C-pentose Total anthocynins Cyanidin-3-rutinoside
**Algeria**	Bakkor Khal Onk Elhamam	(Mahmoudi et al., 2018)	Total phenolic content Total anthocynins Total flavonoid content
**Morocco**	Fassi Noukali	(Hssaini et al., 2020, 2021)	Total phenolic content Total anthocynins

Among the diverse array of *F. carica* varieties, certain varieties have shown exceptional potential. Notably, in the ‘Dottato’ and ‘Cuello de Dama’ varieties from Italy, cyanidin-3-rutinoside, a type of anthocyanin, was identified within the fruit’s pulp (Russo et al., 2014). Additionally, the ‘Boughandjo’, a high-yielding variety, and the ‘Bither’, an early-ripening variety from Algeria, although not the richest in secondary metabolites, have gained consumer favor for their taste (Mahmoudi et al., 2018). Moreover, cluster analysis of 37 F*. carica* varieties has identified ‘Grise de Saint Jean’ and ‘Grise de Tarascon’ as having minimal levels of furanocoumarins (which cause skin inflammation), making them promising candidates for the development of functional foods and pharmaceutical products (Takahashi et al., 2017).

### 3.2 Tissues

Different tissues of *F. carica* have different secondary metabolite profiles (**Table 2**). Previous studies have focused more on the secondary metabolites found in the fruits, pulp, peel, leaves, and latex of *F. carica*, with only a limited number of studies exploring the roots and bark.

**Table 2:** Dominate compounds in different tissues of *F. carica*.

Tissue	Classification	Constituents	Dominant Compounds
**Fruit**	Flavonoids	Flavonols	Quercetin 3-O-rutinoside; Quercetin-3-glucosides
		Anthocyanins	Polymeric Procyanidins; Cyanidin-3-O-rutinoside
		Isoflavones	Ficucaricone A; Ficucaricone B; Ficucaricone C; Ficucaricone D
**Peel**	Flavonoids	Flavonols	Quercetin 3-O-rutinoside; Quercetin-3-glucosides
		Anthocyanins	Polymeric Procyanidins; Cyanidin-3-O-rutinoside
**Pulp**	Flavonoids	Anthocyanins	Cyanidin-3-O-rutinoside
		Isoflavones	Prenylgenistein III; 7methoxy 2’hydroxy genistein (cajanin)
**Leave**	Flavonoids	Flavonols	Quercetin 3-O-rutinoside
	Coumarins		Psoralen; Bergapten
	Terpenes	Sesquiterpenes	β-Caryophyllene; δ-Elemene
**Latex**	Sterols		β-sitosterol; Lupeol; 6-O-acyl-beta-D-glucosyl-beta-sitosterols
**Woody Part**	Coumarins		Psoralen; Bergapten
**Bark**	Flavonoids	Flavonols	Quercetin 3-O-rutinoside
**Root Heartwood**	Coumarins		Psoralen; Bergapten
	Sterols		β-sitosterol

*F. carica* fruits serve as a rich reservoir of flavonoids, which notably include quercetin 3-O-rutinoside, quercetin-3-glucosides, polymeric procyanidins, and cyanidin-3-O-rutinoside among their key constituents (Mopuri and Islam, 2016). Quercetin-3-O-rutinoside (rutin) is the predominant flavonoid found in light-colored *F. carica* peel, whereas cyanidin-3-O-rutinoside is the primary flavonoid present in dark-colored *F. carica* peel (Palmeira et al., 2019; Calani et al., 2022). Within fruits, the peel is richer in secondary metabolites than the pulp. For example, the anthocyanin content of the peel of the dark-colored ‘Mission’ variety was 100-fold that of the pulp (Solomon et al., 2006). Portuguese *F. carica* variety had higher levels of nutritional and phenolic components 158 (Palmeira et al., 2019). Interestingly, an exception is observed in the green *F. carica* varieties ‘Bidhi’ and ‘Kadota’, where the concentration of total phenolics in the edible pulp significantly surpasses that found in the peel (Mahmoudi et al., 2018). However, in the *F. carica* processing industry, peels are frequently discarded as waste, highlighting the importance of studying the bioactivity of *F. carica* peels and the need to develop and utilize by-products.

Notably, the fruit pulp is a significantly richer source of prenylated flavonoids compared to the fruit peel (Ammar et al., 2015; Liu et al., 2019). A study analyzed the phenolic composition of leaves, fruit, peel, and pulp, identifying an isomer of prenylhydroxygenistein as the primary compound in the pulps (Ammar et al., 2015). This compound, a type of prenylated flavonoids, is known for its anti-inflammatory and cancer-preventive activities, making it a valuable constituent for the development of functional foods (Chang et al., 2021).

The bioactive compounds present in *F. carica* leaves were extensively summarized in a previous review (Li et al., 2021). Leaves are found to be more abundant in secondary metabolites compared to their fruit counterparts. Overall, *F. carica* leaves are a good source of flavonoids, coumarins, and terpenes. Rutin is the main flavonoid in *F. carica* leaves, and psoralen is the main furanocoumarin compound (Ammar et al., 2015). An experiment measuring coumarin content in leaves, bark, and the woody part of *F. carica* showed that the leaves contain higher levels of psoralen and bergapten (Conforti et al., 2012). Psoralen and bergapten are also major coumarin compounds in the root heartwood (Jaina et al., 2013). Recent research on the phenolic composition of *F. carica* bark identified rutin as the predominant compound (Yahiaoui et al., 2024). In addition, compared with pulp and peel, *F. carica* leaves contain a notably higher content of sesquiterpenes, with β-Caryophyllene and δ-Elemene as the main components, but monoterpenes are rare (Oliveira et al., 2010c).

Latex is rich in coumarins and has a large amount of sterols. A hexane extract of Tunisian common ‘Jrani caprifig’ latex identified 36 compounds, 14 of which were coumarins (Lazreg-Aref et al., 2012). A total of 7 phytosterols were identified, among which β-sitosterol and lupeol were the compounds with higher concentrations (Oliveira et al., 2010a). Notably, a mixture of 6-O-acyl-beta-D-glucosyl-beta-sitosterols, which has a significant inhibitory effect on cancer cells, was only isolated from latex (Rubnov et al., 2001).

## 4 Factors that affect the secondary metabolite biosynthesis and accumulation

### 4.1 Abiotic stresses

Environmental factors and stressors affect secondary metabolite accumulation in *F. carica*. Light influences the biosynthesis of anthocyanins in plants (Mao et al., 2023). Although light deprivation did not affect the coloration or anthocyanin content of *F. carica* female flower tissues, it significantly inhibited the biosynthesis of anthocyanins in fruit peel (Wang et al., 2019). In the bagged fruit (female flower tissue), 12 flavonoids were detected, of which 11 increased, and apigenin increased by 11 times (Wang et al., 2021). In addition, pulse light treatment using a high-energy xenon flashlight after harvesting resulted in a 20-fold increase in the anthocyanin content of *F. carica* fruit peel. Moreover, pulse light treatment increased the total phenolic content of fruit. Therefore, pulse light markedly promotes the accumulation of anthocyanins and other phenolic compounds in *F. carica* (Rodov et al., 2012).

**Figure 2:**
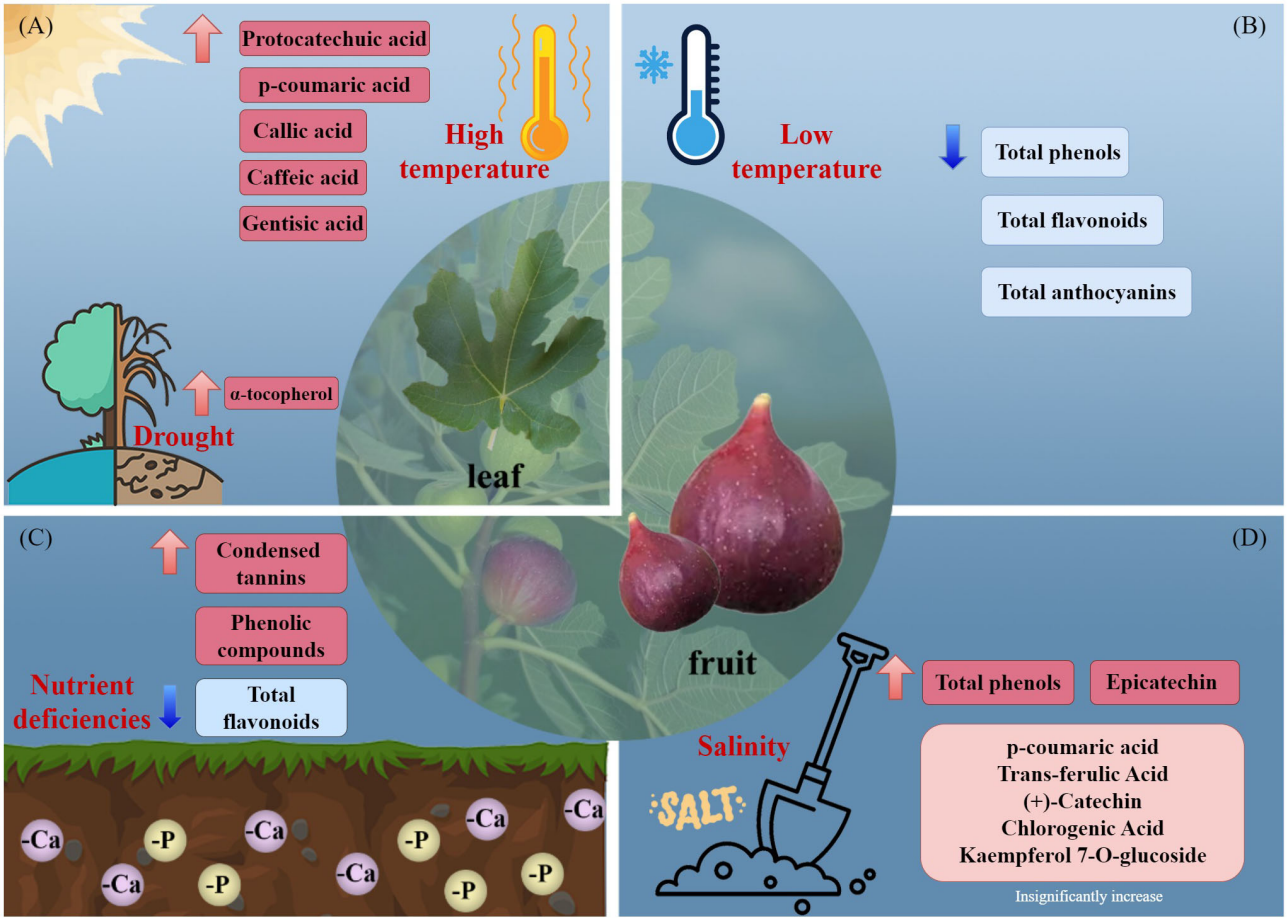
Effects of environmental stresses affect the accumulation of secondary metabolites. **(A)** high temperature and drought, **(B)** low temperature, **(C)** nutrient deficiencies, and **(D)** salinity.

Increased production of secondary metabolites can ameliorate stress-induced damage in plants (**Figure 2**). The total phenolic level of *F. carica* is increased by phosphorus and calcium deficiency (Garza-Alonso et al., 2020). Salt stress increased the total phenolic content of *F. carica* by 5.6%, with a particularly pronounced increase observed in the levels of epicatechin (Francini et al., 2021). Additionally, high temperatures increase the phenolic content of the leaves of *F. carica* seedlings (Guo et al., 2017), and drought stress increases the α-tocopherol content (Gholami et al., 2012). In a study of fresh, frozen, and processed fruit, storage of *F. carica* at −18°for 4 months resulted in significant decreases in the total phenolic, total flavonoid, and total anthocyanin contents (Petkova et al., 2019).

### 4.2 Phytohormone treatments

**Figure 3:**
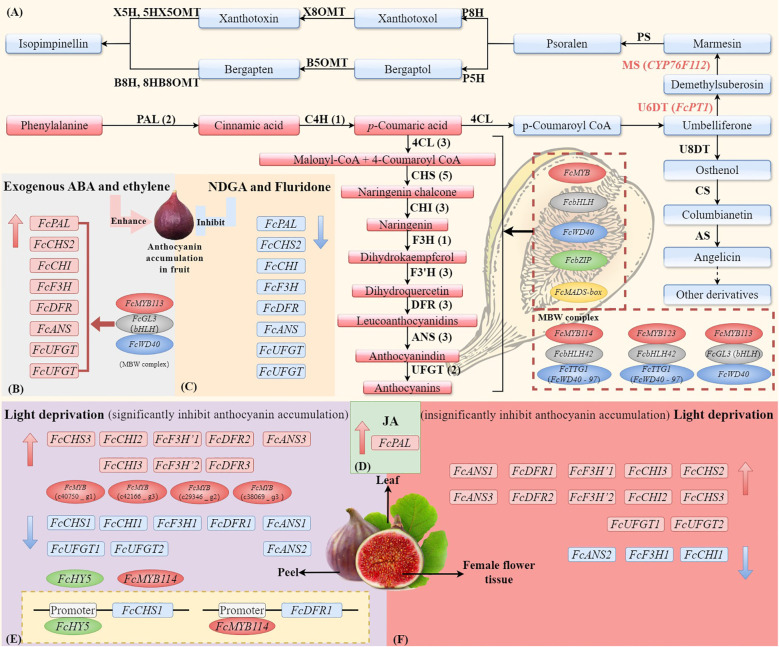
The synthesis pathways of anthocyanin and coumarin in *F. carica* and the external factors (ABA, light, and JA) affect the expression of genes and TFs. **(A)** The biosynthesis of anthocyanins in *F. carica* is regulated by MYB, bHLH, WD40, bZIP, and MADS-box transcription factors, as well as the MBW (MYB-bHLH-WD40) complex. Effects of exogenous abscisic acid **(ABA)** or ethylene **(B)**, ABA inhibitors nordihydroguaiaretic acid (NDGA) or fluridone **(C)**, and jasmonic acids (JAs) **(D)** on the expression of anthocyanin biosynthesis genes in fruit. Effects of light deprivation on anthocyanin biosynthesis genes in fig peel **(E)** and female flower tissue **(F)**. The structural genes involved in the biosynthesis of anthocyanins include phenylalanine ammonium lyase (PAL), cinnamate-4-hydroxylase (C4H), 4-coumarate: coenzyme A ligase (4CL), chalcone synthase (CHS), chalcone isomerase (CHI), flavanone 3-hydroxylase (F3H), flavanone 3’-hydroxylase (F3’H), dihydroflavonol 4-reductase (DFR), anthocyanin synthetase (ANS), and UDP glucose: flavonoid 3-O-glucosyltransferase (UFGT). The simplified biosynthetic pathways of coumarin and furanocoumarin (FC). The core skeleton structures of linear and angular FCs are psoralen and angelicin. The key enzymes involved in the biosynthesis of FCs include umbelliferone 6-dimethylallyltransferase (U6DT), umbelliferone 8-dimethylallyltransferase (U8DT), marmesin synthase (MS), columbianetin synthase (CS), psoralen synthase (PS), angelicin synthase (AS), psoralen 5-hydroxylase (P5H), psoralen 8-hydroxylase (P8H), bergaptol 5-O-methyltransferase (B5OMT), xanthotoxol 8-O-methyltransferase (X8OMT), bergapten 8-hydroxylase (B8H), xanthotoxin 5-hydroxlyase (X5H), 8-hydroxybergapten 8-O-methyltransferase (8HB8OMT), and 5-hydroxyxanthotoxin 5-O-methyltransferase (5HX5OMT).

Phytohormones related to stress resistance, such as abscisic acid (ABA), jasmonic acid, and ethylene, can modulate the biosynthesis of anthocyanins in *F. carica* (**Figure 3**) (Guo et al., 2017; Lama et al., 2019; Cui et al., 2021). ABA promotes the accumulation of anthocyanins during fruit ripening, thereby initiating color development (Lama et al., 2019). *F. carica* treated with ABA had higher levels of cyanidin 3-O-glucoside and cyanidin 3-O-rucoside and developed a dark-purple color earlier compared with those treated with an ABA inhibitor (Lama et al., 2020). Transcriptomic analysis indicated that ABA upregulated the expression levels of genes related to anthocyanin biosynthesis, whereas an ABA inhibitor reversed this effect, confirming the importance of ABA for the biosynthesis of anthocyanins. Ethylene is a phytohormone associated with plant maturation and is implicated in fruit development and stress responses. Ethylene has bidirectional regulatory effects on female flower and peel coloration in *F. carica*. Treatment of *F. carica* with exogenous ethylene upregulated the expression levels of genes related to flavonoid biosynthesis, thereby accelerating the coloration of the receptacle, whereas downregulation of these genes delayed the coloration of female tissues (Cui et al., 2019). In addition, *F. carica* seedlings treated with methyl jasmonate under high-temperature stress had elevated total phenolic contents (Guo et al., 2017).

### 4.3 Developmental factors

Developmental and ripening stages affect the accumulation of secondary metabolites in *F. carica* fruit (Veberic et al., 2008). Some species of *F. carica* can ripen twice and be harvested twice annually: first from late May until the end of June (‘Breba’) and subsequently from mid-July to early September (main crop). In the *F. carica* cultivars ‘Zuccherina’, ‘Crna Petrovka’, and ‘Miljska’, fruits from the second ripening had higher levels of phenolic than those from the first ripening (Veberic et al., 2008). Similarly, the main crops of two ‘Albanian’ cultivars had higher phenolics contents compared with the ‘Brebas’ from the second ripening (Hoxha and Kongoli, 2016). However, in the cultivars ‘VB1’, ‘Antonio’, and ‘Santado’, the levels of phenolic compounds were higher in fruits from the first ripening than in those from the second ripening, suggesting that secondary metabolite accumulation varies among developmental and ripening stages (Vallejo et al., 2012). In addition, the contents of furanocoumarins and pyranocoumarins are higher in the first ripening in June, whereas those of polyphenolics are highest in the second ripening in September (Marrelli et al., 2012).

The types and levels of secondary metabolites also vary according to the maturity of *F. carica* fruit within a ripening stage. Among fruits at the immature, mature, and fully mature stages, the total phenolic content increased during maturation and peaked at the fully mature stage (Pereira et al., 2017). However, others have reported that the total phenolic content decreases with fruit development (Gündeli et al., 2021; Hoxha et al., 2022). In addition, the polyphenolic contents of dark-colored *F. carica* cultivars change more during ripening compared with light-colored cultivars (Wang et al., 2019). Overall, the accumulation of secondary metabolites in *F. carica* fruit is influenced by the developmental time, stage of maturity, and also depends on the cultivars.

### 4.4 Post-harvest processing techniques

As a result of its susceptibility to physical damage and post-harvest infection, fresh *F. carica* fruit is typically processed into dried fruits, jams, jellies, and syrups, as examples. These processes affect the secondary metabolite profiles of *F. carica* fruit (Liu et al., 2020). Drying, including sun- and oven-drying, is the most common method for preserving fresh *F. carica* fruit. Sun drying markedly reduces the levels of polyphenols in fruit, and the effect is greater on flavonoids than on phenolic acids. Sun drying decreased the contents of phenolic acids and flavonoids in fruit by 29% and 86%, respectively (Bachir et al., 2016). Similarly, the total phenolic and total anthocyanin contents of a yellow- and a purple-colored fruit variety decreased significantly after drying, but the total flavonoid and proanthocyanidin contents of the yellow fruit variety significantly increased (Kamiloglu and Çapanoğlu, 2015). However, the levels of phenolic compounds, except apigenin-3-O-rutinoside, were high after oven drying, suggesting that oven drying can increase the total phenolic content and the antioxidant activity of fruit (Slatnar et al., 2011). The processing of fruit pulp into jam and honey also reduces the content of secondary metabolites (Tanwar et al., 2014). Although nectar processing, and jam processing reduce the levels of secondary metabolites, considerable quantities, particularly of carotenoids and phenols, remain after jam processing (Tanwar et al., 2014; Petkova et al., 2019). This suggests that the processing of fruit into jams maintains, at least in part, their contents of phenolics and carotenoids.

## 5 Enzymes involved in secondary metabolism in *F. carica*

The first preliminary genome sequence of *F. carica* was obtained by sequencing the genome of the Japanese traditional variety ‘Horaishi’ based on the Illumina platform. The first high-density linkage map was constructed using restriction enzyme site-associated sequencing (Mori et al., 2017). Further sequencing and assembly of the *F. carica* genome, ‘Dottato’, have been conducted using third-generation sequencing technologies (Usai et al., 2020). This version of the genome assembly of *F. carica* comprises 333 Mbp, 80% of which is anchored to 13 chromosomes (Usai et al., 2020). Later, the newest genome of *F. carica* ‘Orphan’ was built, with a total length of 366.34 Mb and a contig N50 of 9.78 Mb (Bao et al., 2023). Generally, ten families of structural genes associated with the anthocyanin biosynthesis pathway have been identified: *FcPAL* (two genes), *FcC4H* (one gene), *Fc4CL* (three genes), *FcCHS* (five genes), *FcCHI* (three genes), *FcF3H* (one gene), *FcF3’H* (three genes), *FcDFR* (three genes), *FcANS* (three genes), and *FcUFGT* (two genes) (Li et al., 2020) (**Figure 3**).

### 5.1 Chalcone synthase and chalcone isomerase

Chalcone synthase (CHS) is a key enzyme that catalyzes the first committed step in the anthocyanin biosynthesis pathway (Jez and Noel, 2001). As the receptacle tissues of *F. carica* develop and ripen, the contents of flavonoids increase, and the expression level of *FcCHS* is upregulated (Cui et al., 2021). *FcCHS1* encodes 389 amino acid residues, including two phenylalanine residues essential for substrate specificity (Li et al., 2020). In addition, the transcription of *FcCHS1* can be regulated by light-induced signal transduction elements such as HY5 (Wang et al., 2019). Subsequent to the reaction catalyzed by CHS, chalcone isomerase (CHI) catalyzes the conversion of chalcone into naringenin (Ralston et al., 2005). *FcCHI1* encodes a 257 amino-acid type I chalcone isomerase, which includes four conserved active sites (Li et al., 2020). The expression levels of *FcCHS1* and *FcCHI1* increase with the accumulation of anthocyanins and are significantly higher in pigmented tissues (fruit peels and flowers) than non-pigmented tissues. The expression of *FcCHS1* is very low in non-pigmented tissues (Li et al., 2020). *FcCHS1* and *FcCHI1*, together with their homologs associated with anthocyanin accumulation, have similar molecular characteristics and secondary structures, implicating their involvement in *F. carica* fruit coloration.

### 5.2 Dihydroflavonol-4-reductase

Dihydroflavonol-4-reductase (DFR) is a key enzyme involved in the later steps of anthocyanin biosynthesis. It catalyzes the conversion of dihydroflavonols to colorless anthocyanins. Metabolomic and transcriptomic analyses showed that the expression levels of three *FcDFR* genes were significantly increased in fruit peel. Among them, the expression levels of *FcDFR2* and *FcDFR3* were upregulated by light deprivation, whereas *FcDFR1* was downregulated (Wang et al., 2019). *FcDFR1* encodes a 363-amino-acid protein encompassing a typical NADP-binding region and a substrate-binding region (Li et al., 2020). The secondary structure of *FcDFR1* is similar to *MnDFR1* (*Morus notabilis*), which has been implicated in anthocyanin accumulation, suggesting that *FcDFR1* is involved in the anthocyanin biosynthesis of *F. carica*.

### 5.3 Anthocyanidin synthase and UDP-glycose flavonoid glycosyltransferase

Anthocyanidin synthase (ANS) catalyzes the penultimate step in the anthocyanin biosynthesis pathway, the conversion of colorless into colored anthocyanins. *FcANS1* encodes a 358-amino-acid protein encompassing a conserved domain 2OG-FeII-Oxy (Cao et al., 2016). *FcANS1* was significantly upregulated in *F. carica* fruit peel compared with flower tissue (Wang et al., 2019). The expression pattern of *FcANS1* and the anthocyanin content are correlated during fruit development. Bagging treatment resulted in downregulation of *FcANS1* expression in fruit peel and female flowers, suggesting that *FcANS1* functions in anthocyanin biosynthesis (Cao et al., 2016). On the contrary, three *FcANS* genes *FcANS1*, *FcANS2* and *FcANS3* in female flower tissues were upregulated after bagging (Wang et al., 2021).

UFGT is the last key enzyme in the anthocyanin synthesis pathway, which can catalyze the glycosylation of unstable anthocyanins to form stable anthocyanins. The open reading frame of *FcUFGT1* is 1374 bp. The expression level of *FcUFGT1* in the fruit peel of *F. carica* at the mature stage was much higher than that in female flower tissue. However, the expression of *FcUFGT1* was significantly downregulated in the fruit peel by light deprivation but significantly upregulated in the female flower tissue (Wang et al., 2019).

### 5.4 Other enzymes

Glutathione S-transferases (GSTs) are multifunctional enzymes involved in the transport of anthocyanins to vacuoles (Gullner et al., 2018). A total of 53 *GST* genes from five subfamilies are present in the *F. carica* genome. *FcGSTF1* and *FcGSTU5/6/7* may be important in anthocyanin accumulation in *F. carica* peel, but further research is needed (Liu et al., 2023).

Compared to anthocyanins, less research has focused on the regulation of the biosynthesis of other secondary metabolites in *F. carica*. Furanocoumarins (FCs), like anthocyanins, are synthesized via the phenylpropanoid pathway and are structurally classified as angular and linear furanocoumarins (Villard et al., 2019). The first step of the furanocoumarin pathway is catalyzed by the umbelliferone dimethylallyl transferase (UDT) and is important for the prenylation of umbelliferone (**Figure 3**). Comparative RNA-seq and enzyme activity analyses of *F. carica* latex organs have shown that *FcPT1* has UDT activity and participates in the biosynthesis of furanocoumarins in *F. carica*, converting umbelliferone into demethylsuberosin (DMS) (Munakata et al., 2020). However, the functions of these enzymes need to be validated by *in vitro* studies. Marmesin synthase is a key enzyme in the biosynthesis of furanocoumarins and is involved in the production of the defense compound psoralen from p-coumaroyl coenzyme A (Villard et al., 2021). The cytochrome P450 gene *CYP76F112* in *F. carica* encodes marmesin synthase, which can convert DMS into marmesin with high affinity. The discovery of *CYP76F112*, the first identified marmesin synthase, expands the potential applications of the furancoumarin pathway (Villard et al., 2021).

## 6 Transcription factors regulating the biosynthesis of secondary metabolites in *F. carica*

Transcription factors modulate the expression of structural genes associated with the biosynthesis of secondary metabolites. They bind to *cis*-acting regions, thereby inhibiting or promoting gene expression. These factors collaboratively or independently regulate the synthesis of enzymes involved in the biosynthesis of secondary metabolites, such as anthocyanins.

### 6.1 MYB transcription factors

MYB transcription factors are widely distributed in plants and are involved in a variety of aspects of plant growth and metabolism (Pratyusha and Sarada, 2022). Transcriptomic analysis revealed that light deprivation alters the expression of several *F. carica* genes, such as *MYB114* and three R2R3MYB transcription factors, with *FcMYB114* specifically regulating *FcDFR1* and anthocyanin biosynthesis via promoter interaction (Wang et al., 2019). Virus-induced gene silencing of *FcMYB114* led to significant downregulation of *FcDFR1*, *FcANS1*, and *FcUFGT1*, resulting in a reduction of anthocyanin content in *F. carica* fruit peel (Chen et al., 2023). Overexpression of two R2R3-MYB genes, *FcMYB21* and *FcMYB123*, led to significantly increased anthocyanin content in apple peel and fruit callus tissues (Li et al., 2020). Furthermore, the significant promotion of *MdMYC2* expression by overexpressing *FcMYB21* suggests a potential close relationship between *FcMYB21* and *FcMYC2* in promoting *F. carica* fruit coloring.

### 6.2 Basic helix-loop-helix transcription factors

The bHLH family is the second largest group of plant transcription factors involved in plant growth, development, and signal transduction. bHLH transcription factors can interact with MYB transcription factors to regulate the biosynthesis of anthocyanins (Hichri et al., 2010). The genome of *F. carica* contains 118 bHLH genes (Song et al., 2021). According to the bHLH family classification in *Arabidopsis*, *FcbHLH* genes can be classified into 25 subgroups. bHLH genes of different subfamilies show different expression patterns in female flower tissues and fruit peel during *F. carica* fruit development. The transient transformation of *FcbHLH42* in tobacco induced a significant increase in anthocyanin content, suggesting that *FcbHLH42* promotes the accumulation of anthocyanins in *F. carica*. The levels of WD40 and other MYB transcription factors were positively correlated with the expression of *FcbHLH42*, providing insight into the regulatory mechanism of flavonoid biosynthesis in *F. carica* (Song et al., 2021).

### 6.3 WD40

The WD40 transcription factors are structurally stable and important regulators of plant development and physiology, including the biosynthesis of anthocyanins. TTG1 was the first WD40 protein discovered in *Arabidopsis thaliana*, and it forms a trimer with *AtMYB123* and *AtbHLH42* to promote anthocyanin biosynthesis (Baudry et al., 2004). The *F. carica* genome contains a total of 204 WD40 genes. Subcellular localization prediction showed that 109 *FcWD40* proteins were localized to the cytoplasm, 69 to the nucleus, and 26 to other cellular compartments. *FcWD4097* has been identified and named *FcTTG1*. The expression level of *FcTTG1* was significantly highest in the flesh and peel, followed by the stem of *F. carica* (Fan et al., 2022).

MYB transcription factors form the MBW complex by binding to the *FcMYB113*, *FcGL3*, and *FcWD40* proteins of the MYB, bHLH, and WD-repeat families, respectively. This complex directly activates the expression of genes involved in the biosynthesis of anthocyanins (Gonzalez et al., 2008). The MBW complex was identified in pollinated and parthenocarpic *F. carica* fruits. *FcMYB113* was significantly downregulated, whereas *FcGL3* and *FcWD40* were moderately upregulated by exogenous ABA and ethylene. The high expression of *FcMYB113*, *FcGL3*, and *FcWD40* during the later stages of anthocyanin biosynthesis may affect coloration (Lama et al., 2020). In addition, yeast two-hybrid assays and biomolecular fluorescence complementation experiments showed that *FcWD40–97* and *FcbHLH42* regulate the biosynthesis of anthocyanins by forming MBW complexes with *FcMYB114* and *FcMYB123* (Fan et al., 2022).

### 6.4 Other transcription factors

Other families of transcription factors, such as MADS-box and bZIP, have been identified in *F. carica*. MADS-box proteins are functionally diverse and regulate multiple processes, including floral organ development, fruit development and maturation, and seed coloring (Aerts et al., 2018). The *F. carica* genome has a total of 64 *FcMADS-box* genes, the expression levels of which were shown to be tissue specific (Kmeli et al., 2023). *FcMADS9* has been cloned, and its regulation of the biosynthesis of anthocyanins in *F. carica* has been validated by heterologous expression in apple fruit and callus tissues (Li et al., 2021).

The bZIP family is one of the largest gene families in plants and is important for biological processes such as secondary metabolism, stress responses, and seed maturation. Y1 screening indicated that *FcHY5* (bZIP) binds to the promoter region of *FcCHS*, implicating the latter in transcription of the structural genes linked to the biosynthesis of anthocyanins (Wang et al., 2019).

## 7 Concluding remarks and future perspectives

We have conducted a comprehensive review of the functions and biosynthesis of secondary metabolites in the economically significant *F. carica*. A diverse array of secondary metabolites in *F. carica*, such as flavonoids, terpenes, and coumarins, exhibit various beneficial activities. Dark-colored *F. carica* varieties and the fruit peel display elevated levels of secondary metabolites.

To fully exploit the potential application of these metabolites in food and medicine, it is crucial to acquire a more in-depth understanding of their biological activity and pharmacological efficacy. The functions of the flavonoids, terpenoids, and coumarins of *F. carica* require further investigation in cell-culture experiments, animal models, and clinical studies to identify potential health benefits. In addition, while *F. carica* is primarily consumed as fruit, the leaves contain higher levels of coumarin compounds, which are usually discarded as industrial byproducts without utilization; therefore, further research on the use and value of these byproducts is necessary.

The content of secondary metabolites undergoes substantial changes during the development and maturation of *F. carica* fruits, and it is also influenced by the genotype of *F. carica*. Additionally, a range of environmental factors (e.g., light), stresses (e.g., high temperature, low temperature, drought, nutrient deficiencies, salinity), hormone treatments (e.g., ABA, ethylene, NDGA, fluridone, and methyl jasmonate), as well as developmental factors, can affect the accumulation of secondary metabolites in *F. carica*. Post-harvest processing methods are worth further exploration as a low-cost and practical technique to maximize the retention of secondary metabolites in *F. carica*. Some cultivation and management practices can be applied to *F. carica* to achieve better fruit quality, including artificial light supplementation systems, fertilizer rationing, and the application of growth regulators.

Anthocyanins, as secondary metabolites in *F. carica*, play a significant role in determining the fruit’s appearance and nutritional value. The availability of the *F. carica* genome has facilitated the investigation of key genes involved in anthocyanin biosynthesis and their regulatory mechanisms. Transcriptomic and bioinformatics analyses have identified several structural genes, including *FcPAL*, *FcCHS*, *FcCHI*, *FcF3H*, *FcDFR*, *FcANS*, *FcUFGT*, and *FcGST*, which regulate anthocyanin synthesis. Furthermore, *CYP76F112* and *FcPT1* have been identified as crucial enzymes in the coumarin compound synthesis pathway. Moreover, MYB, bHLH, WD40, bZIP, and MADS-box transcription factors are implicated in the regulation of the biosynthesis of anthocyanins. Genetic and molecular studies are needed to further identify the functional genes involved in the regulation of secondary metabolite biosynthesis. The application of genetic transformation systems (root hair transformation, virus-induced gene silencing, and gene editing) will facilitate the development of biotechnological techniques for increased contents of bioactive secondary metabolites in *F. carica* varieties. Studies on the biosynthesis mechanisms of anticancer bioactive compounds such as psoralens and saponins in *F. carica* warrant further research.

## Author contributions

YW: Writing – original draft, Writing – review & editing, Visualization. XL: Writing – review & editing, Visualization. SC: Writing – review & editing. QW: Writing – review & editing. BJ: Writing – review & editing. LW: Conceptualization, Writing – original draft, Writing – review & editing.
